# Advances in topiramate as prophylactic treatment for migraine

**DOI:** 10.1002/brb3.2290

**Published:** 2021-09-02

**Authors:** Chuan Hu, Yixin Zhang, Ge Tan

**Affiliations:** ^1^ Department of Neurology The First Affiliated Hospital of Chongqing Medical University Chongqing China

**Keywords:** migraine, prophylactic treatment, topiramate

## Abstract

It is well‐known that topiramate as a kind of antiepileptic drug has been proved effective for migraine prevention in North America and Europe. However, topiramate is still viewed as an off‐label medication for migraine treatment in China, partly because of the limited evidence in Chinese patients. We summarize the effects of topiramate on the frequency, severity, quality‐of‐life, and adverse event among migraine patients, including children and adolescent in this review, so as to provide reference for Chinese doctors.

## Graphical abstract

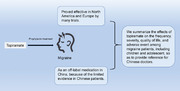

## 1 INTRODUCTION

Migraine is a common neurological disorder that features as a type of recurrent headache lasting 4–72 h, accompanied by nausea, vomiting, and photophobia. The prevalence of migraine in the developed countries is 10%, with 6−8% in male and 12−14% in female (Evers et al., 2009). China shares a similar result the prevalence is 9.3%, with 5.0−6.9% in male and 11.5−14.1% in female (Yu et al., 2012). World Health Organization released a report showing that migraine ranks as the third most prevalent disease and the second most disabling neurological disease in the world (GBD 2016 Disease & Injury Incidence & Prevalence Collaborators, 2017). More than half (51%) of migraineurs are reported to be absent from work or school due to severe headache (Lipton et al., 2001). Moreover, with the frequency of migraine attacks increased, patients’ social burden is significantly increased. In Europe, the annual per capita expenditure on migraine treatment is 1222 Euros (Linde et al., 2012).

Several clinical studies showed a long‐term beneficial role of appropriate preventive treatment for migraine, including improvement in life quality and reduction in health care costs. Thus, it is essential for patients with migraine, especially chronic migraine, to receive a proper prophylactic treatment (GBD 2016 Neurology Collaborators, 2019). Unfortunately, an epidemiologic study conducted in the United States showed that out of approximately 38% of migraine patients that urgently require preventive treatment, only 3−13% of them received a proper treatment (Lipton et al., 2018). Also, a clinic‐based study in China, indicated only 2.7% of migraine patient had been given preventative medication (Li et al., 2012).

Nowadays, a variety of medications are available for migraine prophylaxis, including antiepileptic drugs, calcium channel blockers, beta‐blockers, and antidepressants. Topiramate (TPM), one of the antiepileptic drugs, has been proved to be an effective and well‐tolerated prophylactic medication for migraine patients, and has been officially approved for migraine prophylaxis in the United States. However, unlike other prophylactic medications, TPM is still viewed as an off‐label medication in China, (The Group of Head & Face, Society of Pain, Chinese Medical Association, 2016) partly because of the limited evidence in Asian patients. In this article,we extensively reviewed clinical studies that investigated the effects of TPM on the frequency, severity, and quality‐of‐life among migraine patients, including children and adolescent. Through this review, we expect to provide both theoretical and clinical evidence for Chinese neurologists, especially headache specialists, in TPM prescriptions.

## 2 MECHANISMS OF TOPIRAMATE ON MIGRAINE PREVENTION

**FIGURE 1:**
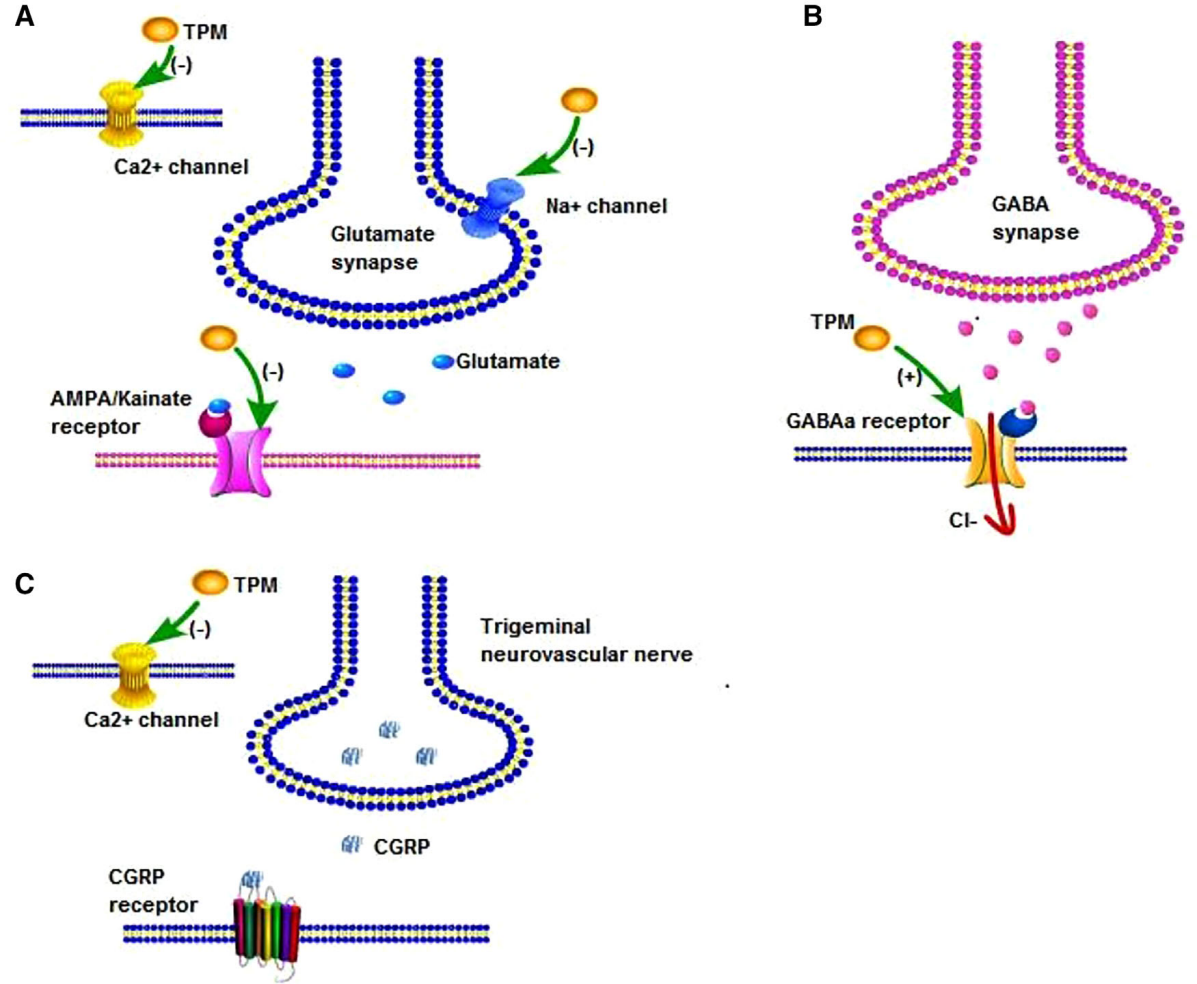
Proposed mechanisms of TPM on migraine prevention. In the preclinical model of seizure, TPM blocks Na^+^ and Ca^2+^ channels (a) on hippocampal, spinal cord neurons and dentate granule cells, then inhibition the release of glutamate via pre‐synaptic mechanism. Besides that, TPM has negative effect on AMPA (a), and positive effect on GABAa receptor (b) to reduce excessive neuronal discharge. In the rat model of migraine and medication overuse headache, TPM reduces the CGRP release (c) via blocking Ca^2+^ channels in trigeminal nerve engdings to prevent the development of CSD and headache.TPM = topiramate; AMPA = aminomethylphosphonic acid; GABAa = gamma‐aminobutyric acid A; CGRP = calcitonin generelated peptide; CSD = cortical spreading depression

TPM was first discovered in 1979 as an intermediate in studies of the treatment of diabetes. It was found to be structurally similar to acetazolamide, a weak carbonic anhydrase isoenzyme inhibitor, that selectively inhibited carbonic anhydrase isoenzymes II and IV (Dodgson et al., 2000). The pathogensis of carbonic anhydrase inhibition in migraine prophylaxis remained unclear, but preclinical studies demonstrated that TPM acts at multiple molecular targets to enhance neuronal inhibition and decrease neuronal excitation (DeLorenzo et al., 2000; Gibbs et al., 2000; McLean et al., 2000; Shank et al., 2000; Skradski & White, 2000; White, 2005; White et al., 1997; Zhang et al., 2000). From many previous studies of animal seizure models in rat and mice, we known that TPM blocks voltage‐activated Na^+^ (DeLorenzo et al., 2000; McLean et al., 2000) and high‐voltage‐activated calcium channels (Zhang et al., 2000) modulates voltage‐gated K^+^ channels to prevent the propagation of action potentials and reduce persistent membrane depolarization (White, 2005), thus, it can stabilize neuronal membrane and reduce neuropeptide release and excessive neuronal discharge by reducing excitability. Second, TPM increases the activity of the gamma‐aminobutyric acid A (GABAa) receptor and thus enhances GABA inhibition (Skradski & White, 2000; White et al., 1997). In addition, unlike other antiepileptic drugs, TPM also negatively modulates aminomethylphosphonic acid (AMPA)/kainate receptors and indirectly inhibits the activity of the N‐methyl‐d‐aspartate (NMDA) receptor (Gibbs et al., 2000), a glutamate receptor found on neurons cells. In the present day, calcitonin gene‐related peptide (CGRP), an important neuropeptide in migraine neurochemistry, is an emerging therapeutic target, and it has been suggested that TPM inhibits the release of CGRP and glutamate from trigeminal neurovascular nerve endings by blocking high‐voltage‐gated Ca^2+^ channels (Dodick, 2018; Durham et al., 2006). Thus, TPM blocks cortical spreading depression (CSD) and has been shown to be effective in an anticonvulsant and antimigraine drug (Dodick, 2018) (Figure 1).

TPM has several potential mechanisms of action on migraine, and the exact mechanism by which it is effective in migraine treatment is not yet known. Based on the results of the animal and cellular studies described above, it is suggested that TPM can have an effect through the opening of ion channels, leading to a reduction in the release of excitatory neurotransmitters, thus negatively regulating neuronal excitability and ultimately relieving migraine, so does TPM have a similar effect in humans? What dose and timeframe is needed to achieve this effect? Many clinical studies in the follow‐up have further elaborated on the emergence of TPM as one kind of prophylactic treatment agent for migraine.

## 3 EFFICACY OF TPM IN MIGRAINE PREVENTION

Topiramate was initially widely used as an anticonvulsant for the treatment of epilepsy, and several randomized controlled trials had recently proven the efficacy of topiramate on migraine prophylaxis.

### 3.1 Effects of TPM on migraine frequency

There was growing interest in the prophylactic use of TPM in migraine patients through a number of randomized controlled clinical trials. Overall effectiveness rates of TPM on migraine attacks (defined as a ≥ 50% reduction in monthly headache attack frequency) ranged from 26 (Storey et al., 2001) to 63% (Gupta et al., 2007) (Table 1). We provide further detailed results from some population‐based control studies below.

**TABLE 1:** Summary of major clinical trial for topiramte on migraine frequency

Researchers	*N*	Design	Time	Participants	Drug	Main results
Silberstein et al. (the MIGR‐001 Study)	487	randomized, double‐blind, placebo‐controlled	26 weeks	EM	topiramate 50, 100, 200 mg/day; placebo	≥ 50% reduction in monthly headache frequency: topiramate group(50 mg/day, 35.9% [*p* = 0.04]; 100 mg/day, 54.0% [*p* < 0.001]; and 200 mg/day, 52.3% [*p* < 0.001]) versus placebo (22.6%).
Brandes et al. (the MIGR‐002 Study)	483	randomized, double‐blind, placebo‐controlled	26 weeks	EM	topiramate 50, 100, 200 mg/day; placebo	Responder rate (compared with placebo, 23%): topiramate at 50 mg/day (39%, *p* = 0.01), 100 mg/day (49%, *p* < 0.001), and 200 mg/day (47%, *p* < 0.001).
Diener et al.	575	randomized, double‐blind, placebo‐controlled	26 weeks	EM	topiramate 100, 200 mg/day; propranolol 160 mg/day; placebo	Responder rate: topiramate 100 mg/day (37%, *p* = 0.01), 200 mg/day (35%, *p* = 0.028) vs placebo (22%).
Gupta et al. (Lotolamp Study)	57	randomized, double‐blind, crossover	20 weeks	EM	topiramate 50 mg/day; lamotrigine 50 mg/day; placebo	Responder rate for frequency: topiramate versus placebo (63% vs 30%, *p* < 0.001), and versus lamotrigine (63 vs 46 %, *p* = 0.019)
Shaygannejad et al.	64	randomized, double‐blind, crossover	24 weeks	EM	topiramate 50 mg/day; valproate 400 mg/day	Reduction of monthly migraine frequency (SD): topiramate from 5.4(2.5) to 4.0(2.8); valproate from 5.4(2.5) to 4.0(2.8)(*p* < 0.001).
Silberstein et al.	306	randomized, double‐blind, placebo, parallel‐group	16 weeks	CM	topiramate; placebo	The mean final topiramate dose was 86.0 mg/day. Mean percentage reduction in the mean number of migraine/migrainous days from baseline compared with placebo (37.1% vs 26.0%, *p* = 0.01).

EM = episodic migraine; CM = chronic migraine; SD = standard deviation.

Two randomized, double‐blind, multicenter, controlled trials in North America evaluated the efficacy of different doses of topiramate for migraine patients over a 26‐week period, comparing 50, 100, and 200 mg topiramate groups with placebo group. These two studies enrolled patients who fulfilled the International Headache Society (IHS) criteria for episodic migraine, and target patients should have a definite history of migraine with or without aura for at least 6 months prior to these trials. Patients were required to have 3–12 migraine episodes at baseline prior to inclusion but no more than 15 headache days per month (28 days). Noteworthy exclusion criteria were previous failure of at least two full courses of migraine prophylaxis regimens, participation in topiramate‐related studies, or taking TPM for more than 2 weeks, and a history of overuse of analgesics or specific medications to acutely treat headache attacks (> 8 treatment days per month of ergots or triptans, or > 6 treatment days per month of powerful opioids). Silberstein et al. (2007) showed that the proportion of patients responsive to TPM (defined as at least a 50% reduction in monthly migraine frequency) was significantly higher in topiramate‐related group than the placebo group, and the proportion reached highest at 100 mg/day group (50 mg/day [35.9%, *p* = 0.04], 100 mg/day [54.0%, *p* < 0.001], 200 mg/day [52.3%, *p* < 0.001], and placebo [22.6%]). Brandes’ trial showed smiliar results: 39% (50 mg, *p* < 0.01), 49% (100 mg, *p* < 0.001) and 47% (200 mg, *p* < 0.001), and 23% (placebo) (Brandes et al., 2004; Diener et al., 2004). Moreover, a European study supervised Christoph Diener also showed 100 mg/day TPM was significantly superior to placebo (37 vs 22%, *p* = 0.01), and the efficacy of TPM on migraine attacks was comparable in patients in 100 mg/day (37%) and 200 mg/day (35%) TPM groups (Diener et al., 2004; Mei et al., 2004). It was found that the above multicenter randomized clinical trials were conducted among patients with episodic migraine aged 12–65 years from North America and Europe. The results of the trial showed that topiramate was significantly more effective than placebo in the preventive treatment of episodic migraine and was comparable to propranolol.

In 2007, a randomized, double‐blind, placebo‐controlled, phase 4 crossover trial (Lotolamp study) conducted by Gupta et al. (2007) was to evaluate low‐dose topiramate (50 mg/day) versus lamotrigine for the prophylactic treatment of frequent migraines (more than four attacks per month). It allocated 60 patients with migraine randomly to receive TPM (25 mg bid) or lamotrigine 50 mg or matching placebo for 1 month in four phases with two crossover doses, separated by a 1‐week washout period. The responsive rate of TPM versus placebo was 63% versus 30% (*p* < 0.001), and for lamotrigine versus placebo was 46 and 34% (*p* < 0.093), but the responder rate for topiramate versus lamotrigine was 63% versus 46% (*p* < 0.019) (Gupta et al., 2007). A similar 24‐week study conducted by Shaygannejad et al. (2006) reported that 50 mg topiramate and 400 mg valproate on migraine prevention were equivalent. Of the 32 patients treated with sodium valporate, the mean standard deviation of monthly migraine frequency decreased from 5.4(2.5) to 4.0(2.8) episode per month (*p* < 0.001). Correspondingly, in the 32 patients treated with TPM, the mean standard deviation of monthly headache frequency decreased from 5.4(2.0) to 3.2(1.9) per month (*p* < 0.001) (Shaygannejad et al., 2006). Compared with other antiepileptic drugs (such as lamotrigine and valproate), some small sample clinical studies had shown that low dose topiramate also had good efficacy.

As for chronic migraine, only one large randomized, double‐blind, placebo‐controlled clinical trial had studied the efficacy and safety of TPM on chronic migraine (more than 15 days of headaches per month, at least half of which are migraine‐like attacks). (Anon, 2018) Compared with placebo group, patients treated with 100 mg/day TPM had remarkable reduction in migraine frequency after a 16‐week period (37.1 vs 26.0%, *p* = 0.01) (Silberstein et al., 2007). These trials clearly documented its efficacy as a migraine preventive drug.

### 3.2 Effects of TPM on migraine severity and quality of life

**FIGURE 2:**
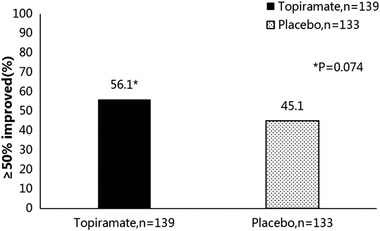
Migraine disability assessment responder analysis, percent of responders

In addition to the significant improvement in migraine attack frequency, TPM also significantly improved overall headache severity, as well as daily living capacity and overall quality of life. In 2007, a study conducted by the Department of Neurology at the Mayo Clinic College of Medicine evaluated the effects of 100 mg TPM on migraine‐related disability and daily activities (Dodick et al., 2007). This investigation was the first reported study of a large, randomized, double‐blind, placebo‐controlled trial in which a total of 328 patients with chronic migraine were randomized (TPM, *n* = 165; placebo, *n* = 163), and showed that the Migraine Disability Assessment Scale (MIDAS) scores in TPM treated group improved by more than 50% from baseline, however, the results reached borderline significantly difference when compared with placebo group (TPM 56 vs placebo 45%; *p* = 0.074) (Figure 2). The Migraine‐Specific Quality of Life Questionnaire (MSQ) analysis showed significant improvements at week 4 in all three domains (Role Function‐restrictive [RFR], Role Function‐preventive [RFP], Emotional Function [EF]), and at week 8 and 16 both RFR and EF domains(Table 2). On the patients’ overall change in clinical condition, 75 and 72% of topiramate‐treated patients versus 61 and 59% of placebo‐treated patients reported improvements on the subject's global impression of change (SGIC) and physician's global impression of change (PGIC) scales (*p* = 0.025 and *p* = 0.037, respectively)(Table 3). Compared with placebo‐treated patients, topiramate 100 mg/day appears to contribute to reductions in migraine‐related limitations on daily activities and emotional distress caused by migraine beginning as early as week 4 and continuing up to week 16 after treatment.

**TABLE 2:** Least squares (LS) mean change from baseline of Migraine‐Specific Quality of Life Questionnaire Role Function Restrictive (RFR), Role Function Preventive (RFP) and Emotional Function (EF)(analysis of covariance, last observation carried forward)

	RFR			RFP			EF		
Time	Topiramate	Placebo	*p*	Topiramate	Placebo	*p*	Topiramate	Placebo	*p*
Week 4	21.7	12.7	＜0.05	14	10.1	＜0.05	25.7	15.2	＜0.05
Week 8	23.6	17.4	＜0.05	15.7	11.8	NS	25.9	19	＜0.05
Week 12	23.8	19.5	NS	16.9	13.1	NS	26.7	20.5	NS
Week 16	24.3	18.5	＜0.05	16.9	12.5	NS	26.9	20	＜0.05

NS = not statistically significant.

**TABLE 3:** Differences in SGIC and PGIC between Topiramate Group and Placebo Group

Instrument	Topiramate (*n* = 153) *N* (%)	Placebo (*n* = 153) *N* (%)	*p*‐value
SGIC			
Improved	106(75.2)	83(60.6)	0.025
Not improved	35(24.8)	54(39.4)	
PGIC			
Improved	102(72.3)	81(58.7)	0.037
Not improved	39(27.7)	57(41.3)	

SGIC = Subject's Global Impression of Change; PGIC = Physician's Global Impression of Change.

### 3.3 Effects of TPM on migraine in children and adolescent

To date, there were limited treatment options for migraine among children or adolescents patients (Table 4). In 2005, Winner et al. from Palm Beach Headache Centre, United States, evaluated the efficacy and safety of TPM for migraine prevention in children (6–15 years of age) in a randomized, double‐blind, placebo‐controlled trial. One‐hundred sixty‐two children were randomized into TPM group (*n* = 112) or placebo (*n* = 50), with a starting dose of 5 mg/day TPM, titrated to 2–3 mg/kg per day, or the maximum tolerated dose (maximum allowable dose of 200 mg/day) over 8 weeks. The target dose of TPM had been maintained for 12 weeks. Intention‐to‐treat analysis showed that 157 (97%) (109 in the topiramate group and 49 in the placebo group) patients included in the intention‐to‐treat analysis received topiramate versus placebo for a mean reduction in headache days per month of 2.6 and 2.0 migraine days, respectively (*p* = 0.061), but this reduction approached statistical significance. In the double‐blind period, the percentage of patients experienced with a ≥50% reduction in migraine days per month was 54.6% in TPM group compared with 46.9% in the placebo group (*p* = 0.39). And the percentage of patients experienced with a ≥ 75% reduction in migraine days per month was significantly higher in TPM group (32.4%) than placebo group (14.3%, *p* = 0.02). (Winner et al., 2005) Another one randomized, double‐blind, placebo‐controlled study of migraine in adolescents (12–17 years old) at Children's Hospital of the King's Daughters in the United States, showed that patients who received 100 mg/day TPM had significant lower frequency in migraine attacks when compared with patients received placebo in the last 12‐week of double blind period (median: 72.2 vs 44.4%, *p* = 0.016). No significant difference was detected between patients who received 50 mg/day TPM and placebo (median: 44.6 vs 44.4%, *p* = 0.798). The above data demonstrated that the most effective TPM dosage in children and adolescent was similar as that in adults (Lewis et al., 2009).

**TABLE 4:** Major clinical trials of TPM on migraine in children and adolescent

Researchers	N	Design	Time	Participants	Drugs	Main results
Winner et al.	162	randomized (topiramate: placebo = 2:1), double‐blind, placebo‐control	20 weeks	Children (6—15 years)	Topiramate (2–3 mg/kg/day); placebo	1.Mean reduction in migraine days per month: topiramate 2.6 versus placebo 2.0 *p p* = 0.061). 2. ≥50% reduction in migraine days per month: topiramate (54.6%) compared with placebo (46.9%) (*p* = 0.39). 3. ≥75% reduction in migraine days per month: significantly higher in topiramate group (32.4%) than placebo group (14.3%, *p* = 0.02)
Lewis D, Winner P et al.	106	randomized, double‐blind, placebo‐control, parallel‐group	16 weeks	Adolescents (12–17 years)	topiramate 50, 100 mg/day; placebo	Reduction in the monthly migraine attack rate: 50 mg/day, 100 mg/day topiramate versus placebo (median: 44.6 vs 44.4%, *p* = 0.798; 72.2 vs 44.4%, *p* = 0.016)
Powers et al.	361	randomized, double‐blind, placebo‐control	24 weeks	children and adolescents (8–17y)	topiramate (2 mg/kg/d); amitriptyline (1 mg/kg/d); placebo	1. ≥50% Reduction of the number of headache days (vs placebo (61%): topiramate (55%), *p* = 0.48; amitriptyline (52%) versus placebo, *p* = 0.26; amitriptyline versus topiramate, *p* = 0.49. 2. Patients completed the trial (95% CI, %): topiramate 78% (71–85), amitriptyline 80% (73–86), placebo 89% (80–95). 3. AE of topiramate (≥10%): paresthesia (31%), fatigue (25%), dry mouth (18%), memory impairment (17%), aphasia (16%), cognitive disorder (16%), and upper respiratory tract infection (12%).

CI = confidence interval; AE = adverse events.

However, the childhood and adolescent migraine prevention (CHAMP) trial (Powers et al., 2017) led by Cincinnati Children's Hospital Medical Centre showed opposite results. They recently enrolled 361 children and adolescents migraine patients treated with topiramate (2 mg/kg per day), amitriptyline (1 mg/kg per day) and placebo. After 24‐week treatment, Powers et al. (2017) found that both TPM and amitriptyline were less effective than placebo on reduction of the headache days after a 24‐week treatment (55% for topiramate, 52% for amitriptyline, 61% for placebo; topiramate vs placebo, *p* = 0.48; amitriptyline vs placebo, *p* = 0.26; amitriptyline vs topiramate, *p* = 0.49). Compared with placebo‐treated patients, TPM and amitriptyline had no significant benefit in migraine patients aged 8–17 years. Also, there was no significant difference between‐groups in terms of headache‐related disability or the percentage of patients completed the 24‐week treatment period. Patients treated with either topiramate or amitriptyline had higher rates of adverse events than those treated with placebo, including abnormal sensations (31 vs 8%, *p <* 0.001) and weight loss (8 vs 0%, *p* = 0.02) in the topiramate group, and fatigue (30 vs 14%, *p* = 0.02) and dry mouth (25 vs 12%, *p* = 0.02) in the amitriptyline group. It was showed that topiramate is a limited and controversial in the treatment of migraine for children and adolescents. There have been fewer clinical trials of topiramate for pediatric migraine compared to adults. Therefore, topiramate should be used with caution in children and adolescent patients.

### 3.4 Safety and tolerability of TPM in migraine treatment

The common adverse events (AEs) associated with topiramate were paresthesia, fatigue, anorexia, upper respiratory tract infection, nausea, diarrhoea, cognitive impairment(memory difficulties, poor concentration, etc.), and weight loss (Table 5). Especially, the topiramate‐associated AEs were usually mild and moderate in severity, and occured in the titration period compared with the maintenance period. Adelman et al. (2008) analyzed the safety and tolerability of 1580 migraine patients enrolled in three key clinical trials.The pooled population consisted of all patients who took at least one study drug during the double‐blind period(topiramate 50 mg/day(*N* = 235), topiramate 100 mg/day (*N* = 386), topiramate 200 mg/day (*N *= 514), or placebo (*N* = 445)). The incidence of discontinuation due to AEs in the topiramate group across all doses was higher during the titration period (23%) than during the maintenance period of the doubleblind phase (5%). The percentages of patients who withdrew due to AEs during the titration period were 16, 23, and 26% of the topiramate 50, 100, and 200 mg/day groups, respectively, and during the maintenance period, 4, 4, and 8%, respectively. The most common event leading to dose reduction or temporary interruption of study medication among topiramate‐treated patients was paresthesia, occurring in 5, 6, and 7% of patients in the topiramate 50, 100, and 200 mg/day groups, respectively. For the recommended dose of topiramate 100 mg/day, the AEs leading to withdrawal were paresthesia (8%), fatigue (5%), nausea (2%), and difficulty with concentration (2%). Serious AEs were rare, occurring in 2% of the 1135 patients treated with topiramate. (Adelman et al., 2008)

**TABLE 5:** Some common adverse events of topiramate groups and placebo group

WHO‐ART preferred term	Placebo *N *= 445	Led to withdrawal	TPM 50 mg/day *N* = 235	Led to withdrawal	TPM 100 mg/day *N* = 386	Led to withdrawal	TPM 200 mg/day *N* = 514	Led to withdrawal	TPM *N* = 1135
Paresthesia	26(6)*	3(1)	83(35)	8(3)	195(51)	31(8)	254(49)	37(7)	534(47)
Fatigue	50(11)	4(1)	33(14)	7(3)	58(15)	18(5)	98(19)	24(5)	189(17)
Anorexia	27(6)	2(< 1)	22(9)	2(1)	56(15)	8(2)	72(14)	14(3)	150(13)
Upper respiratory tract infection	54(12)	0	31(13)	0	54(14)	0	62(12)	0	147(13)
Nausea	37(8)	5(1)	21(9)	7(3)	51(13)	9(2)	73(14)	29(6)	145(13)
Diarrhea	19(4)	2(< 1)	20(9)	2(1)	43(11)	6(2)	54(11)	10(2)	117(10)
Dizziness	44(10)	7(2)	19(8)	2(1)	33(9)	8(2)	62(12)	13(3)	114(10)
Weight decrease	6(1)	0	13(6)	1(< 1)	35(9)	4(1)	58(11)	6(1)	106(9)
Difficulty with concentration/attention	10(2)	1(< 1)	7(3)	1(< 1)	23(6)	8(2)	51(10)	24(5)	81(7)

WHO‐ART = World Health Organization adverse reaction terminology; TPM = topiramte.

* Values expressed as *N* (%).

An individual subject might have experienced more than one treatment‐limiting adverse event.

## 4 CURRENT CONTROVERSIES AND PROSPECTS

Topiramate had become the most commonly used migraine prophylaxis because of the overwhelming evidence of its ability to improve outcomes for migraine patients in terms of reduced disability and improved quality of life, as well as reduced use of social health resources. However, it is clear that disparities still exist, not only in the age of adults versus children and adolescents, differences in efficacy with topiramate and other prophylactic medications, but also in ethnicity, particularly the European and Chinese populations, such as differences in the dosage and duration of topiramate prophylaxis for migraine. In 2007, a 12‐week multicenter prospective observational study in Hong Kong, China, showed that a low daily dose of 25 mg of topiramate was equally effective as 50, 75, and 100 mg in reducing headache frequency (Li et al., 2007). And a similar small sample study in 2010 concluded that low doses (< 100 mg/day) may be effective in the Chinese population (Lo et al., 2010). According to the 2016 Chinese Guidelines for migraine Prevention and Treatment, topiramate is used for migraine prophylaxis at doses of 25–100 mg/day for 3–6 months of prophylactic treatment (The Group of Head & Face, Society of Pain, Chinese Medical Association, 2016). While the American Academy of Neurology/American Headache Society (AAN/AHS) evidence‐based guidelines consider that topiramate is a proven effective migraine prophylactic agent as grade A recommendation, and the recommended dosages of TPM is 50–200 mg/day for 6 months of treatment (Holland et al., 2012). It is also recommended as a grade A drug for migraine prophylaxis in European Federation of Neurological Societies(EFNS) clinical guidelines, with a recommended dose of 25–100 mg/day (Evers et al., 2009).

Since 1988, the diagnosis and treatment of migraine have evolved considerably in terms of both acute and prophylactic treatments, and various treatment options had been emerged to headache specialists. Unfortunately, a large amount of patients who could be potentially benefited from migraine prophylaxis did not receive appropriate treatment. We can find that there were few clinical studies about topiramate prophylaxis and treatment of migraine in the past 10 years, especially in China, and clinical studies on the dosage, duration, effectiveness, safety and other aspects of topiramate are still lacking and inadequate in China compared with those in the European Union and the North of America. Therefore, it is necessary to conduct multicenter clinical trials on topiramate in Asia, especially in Chinese population, so as to provide more adequate clinical evidence for Chinese neurological headache specialists.

## 5 SUMMARY

Topiramate has shown significant improvements in migraine prophylaxis on headache frequency, headache severity, and life outcomes in adults, although the evidence of its efficacy in children and adolescents is lacking. The further clinical study about Chinese migraine patients need to be conducted in the near future.

## CONFLICT OF INTEREST

The authors declare that there is no conflict of interest.

### PEER REVIEW

The peer review history for this article is available at https://publons.com/publon/10.1002/brb3.2290
